# Molecular imaging predicts lack of T-DM1 response in advanced HER2-positive breast cancer (final results of ZEPHIR trial)

**DOI:** 10.1038/s41523-023-00610-6

**Published:** 2024-01-06

**Authors:** Magdalena Mileva, Elisabeth G. E. de Vries, Thomas Guiot, Zéna Wimana, Anne-Leen Deleu, Carolien P. Schröder, Yolene Lefebvre, Marianne Paesmans, Sigrid Stroobants, Manon Huizing, Philippe Aftimos, Jolien Tol, Winette T. A. Van der Graaf, Wim J. G. Oyen, Danielle J. Vugts, C. Willemien Menke-van der Houven van Oordt, Adrienne H. Brouwers, Martine Piccart-Gebhart, Patrick Flamen, Géraldine Gebhart

**Affiliations:** 1 Department of Nuclear Medicine, Institut Jules Bordet, Hôpital Universitaire de Bruxelles (H.U.B), Université Libre de Bruxelles (ULB), Brussels, Belgium; 2 Department of Medical Oncology, University of Groningen, University Medical Center Groningen, Groningen, The Netherlands; 3 Department of Medical Physics, Institut Jules Bordet, Hôpital Universitaire de Bruxelles (H.U.B), Université Libre de Bruxelles (ULB), Brussels, Belgium; 4 Department of Radiopharmacy, Institut Jules Bordet, Hôpital Universitaire de Bruxelles (H.U.B), Université Libre de Bruxelles (ULB), Brussels, Belgium; 5 Department of Medical Oncology, Antoni van Leeuwenhoek-Netherlands Cancer Institute, Amsterdam, The Netherlands; 6 Department of Radiology, Institut Jules Bordet, Hôpital Universitaire de Bruxelles (H.U.B), Université Libre de Bruxelles (ULB), Brussels, Belgium; 7 Data Center, Institut Jules Bordet, Hôpital Universitaire de Bruxelles (H.U.B), Université Libre de Bruxelles (ULB), Brussels, Belgium; 8 Department of Nuclear Medicine, Antwerp University Hospital, Edegem, Belgium; 9 Department of Medical Oncology, Antwerp University Hospital, Edegem, Belgium; 10 Department of Medical Oncology, Institut Jules Bordet, Hôpital Universitaire de Bruxelles (H.U.B), Université Libre de Bruxelles (ULB), Brussels, Belgium; 11 Department of Internal Medicine, Jeroen Bosch Ziekenhuis, Den Bosch, The Netherlands; 12 Department of Medical Oncology, Radboud University Medical Centre, Nijmegen, The Netherlands; 13 Humanitas Clinical and Research Center, Humanitas University, Milan, Italy; 14 Department of Radiology and Nuclear Medicine, Rijnstate Hospital, Arnhem, The Netherlands; 15 Department of Radiology and Nuclear Medicine, Radboud University Medical Center, Nijmegen, The Netherlands; 16 Department of Radiology and Nuclear Medicine, Amsterdam UMC, Vrije Universiteit, Cancer Center Amsterdam, Amsterdam, The Netherlands; 17 Department of Medical Oncology, Amsterdam UMC Location VUMC, Cancer Centre Amsterdam, Amsterdam, The Netherlands; 18 Department of Nuclear Medicine and Molecular Imaging, University of Groningen, University Medical Center Groningen, Groningen, The Netherlands

**Keywords:** Breast cancer, Cancer imaging

## Abstract

Efficacy of the human epidermal growth factor receptor (HER)2-targeting trastuzumab emtansine (T-DM1) in breast cancer (BC) relies on HER2 status determined by immunohistochemistry or fluorescence in-situ hybridization. Heterogeneity in HER2 expression, however, generates interest in “whole-body” assessment of HER2 status using molecular imaging. We evaluated the role of HER2-targeted molecular imaging in detecting HER2-positive BC lesions and patients unlikely to respond to T-DM1. Patients underwent zirconium-89 (^89^Zr) trastuzumab (HER2) PET/CT and [^18^F]-2-fluoro-2-deoxy-D-glucose (FDG) PET/CT before T-DM1 initiation. Based on ^89^Zr-trastuzumab uptake, lesions were visually classified as HER2-positive (visible/high uptake) or HER2-negative (background/close to background activity). According to proportion of FDG-avid tumor load showing ^89^Zr-trastuzumab uptake (entire/dominant part or minor/no part), patients were classified as HER2-positive and HER2-negative, respectively. Out of 265 measurable lesions, 93 (35%) were HER2-negative, distributed among 42 of the 90 included patients. Of these, 18 (19%) lesions belonging to 11 patients responded anatomically (>30% decrease in axial diameter from baseline) after three T-DM1 cycles, resulting in an 81% negative predictive value (NPV) of the HER2 PET/CT. In combination with early metabolic response assessment on FDG PET/CT performed before the second T-DM1 cycle, NPVs of 91% and 100% were reached in predicting lesion-based and patient-based (RECIST1.1) response, respectively. Therefore, HER2 PET/CT, alone or in combination with early FDG PET/CT, can successfully identify BC lesions and patients with a low probability of clinical benefit from T-DM1.

## Introduction

Breast cancer is the most commonly diagnosed malignancy and the leading cause of cancer death in females, with more than 2 million new patients diagnosed worldwide each year^1^. In ~20% of the cases, amplification of the human epidermal growth factor receptor 2 (HER2) gene results in the overexpression of HER2, a transmembrane oncoprotein found on the surface of the cancer cells^2^. Many targeted anti-HER2 agents have been successfully developed in the last 20 years for advanced and early HER2-positive breast cancer. Trastuzumab emtansine (T-DM1) is an antibody-drug conjugate (ADC), incorporating the HER2-targeted antitumor properties of the monoclonal antibody trastuzumab with the cytotoxic activity of the microtubule-inhibitory agent emtansine, both conjugated by a stable linker^3^. It was initially approved to treat patients with advanced HER2-positive breast cancer who progressed after a prior line of trastuzumab-based therapy^4^. More recently, following the randomized phase III clinical trial KATHERINE results, its approval has been expanded to the adjuvant setting to treat patients with HER2-positive early breast cancer with residual invasive disease after neoadjuvant therapy^5^.

To achieve its proper antitumor effect, T-DM1 relies on strong HER2 expression on the tumor cells, which is routinely assessed by immunohistochemistry status (IHC3+) often combined with fluorescence in-situ hybridization (FISH) [*HER2* gene copy number of six or more or a *HER2/CEP17* ratio of 2.0 or greater]^6^. However, HER2 expression can be heterogeneous within an individual tumor or between different tumor lesions, both primary and metastatic^7^, or it can change during the course of the disease from the primary tumor to relapse^8^. Repeated biopsies are recommended to assess the current molecular profile of the tumor in case of disease relapse^9^. The procedure’s invasiveness, along with its inability to reflect the status of the entire tumor load, calls for superior biomarkers to predict response to T-DM1 and better select patients who would benefit most from it. Positron emission tomography/computed tomography (PET/CT) after administration of zirconium-89 (^89^Zr) labeled trastuzumab allows the detection of tumor lesions overexpressing HER2 in patients with HER2-positive breast cancer^10^, potentially serving as a complementary tool for treatment decision making and patient management^11^. Additionally, PET imaging with [^18^F] 2-fluoro-2-deoxy-D-glucose (FDG) has been successfully used to predict the likelihood of response and clinical benefit to treatment in patients with early-stage HER2-positive breast cancer^12,13^.

Therefore, we conducted a study in which patients with advanced HER2-positive breast cancer underwent both ^89^Zr-trastuzumab (HER2) PET/CT and FDG PET/CT before the first administration of T-DM1, followed by an early FDG metabolic response assessment performed just before the second treatment cycle (ZEPHIR study). Our main objective was to evaluate the ability of HER2 PET/CT to predict, before initiation of treatment, tumor lesions unlikely to respond anatomically to T-DM1. The ability of HER2 PET/CT to predict metabolic response after three cycles of T-DM1 was also explored. In addition, we analyzed how an early FDG PET/CT, alone or combined with the pre-treatment HER2 PET/CT, can identify tumor lesions that will not respond (anatomically and metabolically) after three T-DM1 cycles.

**Fig. 1:**
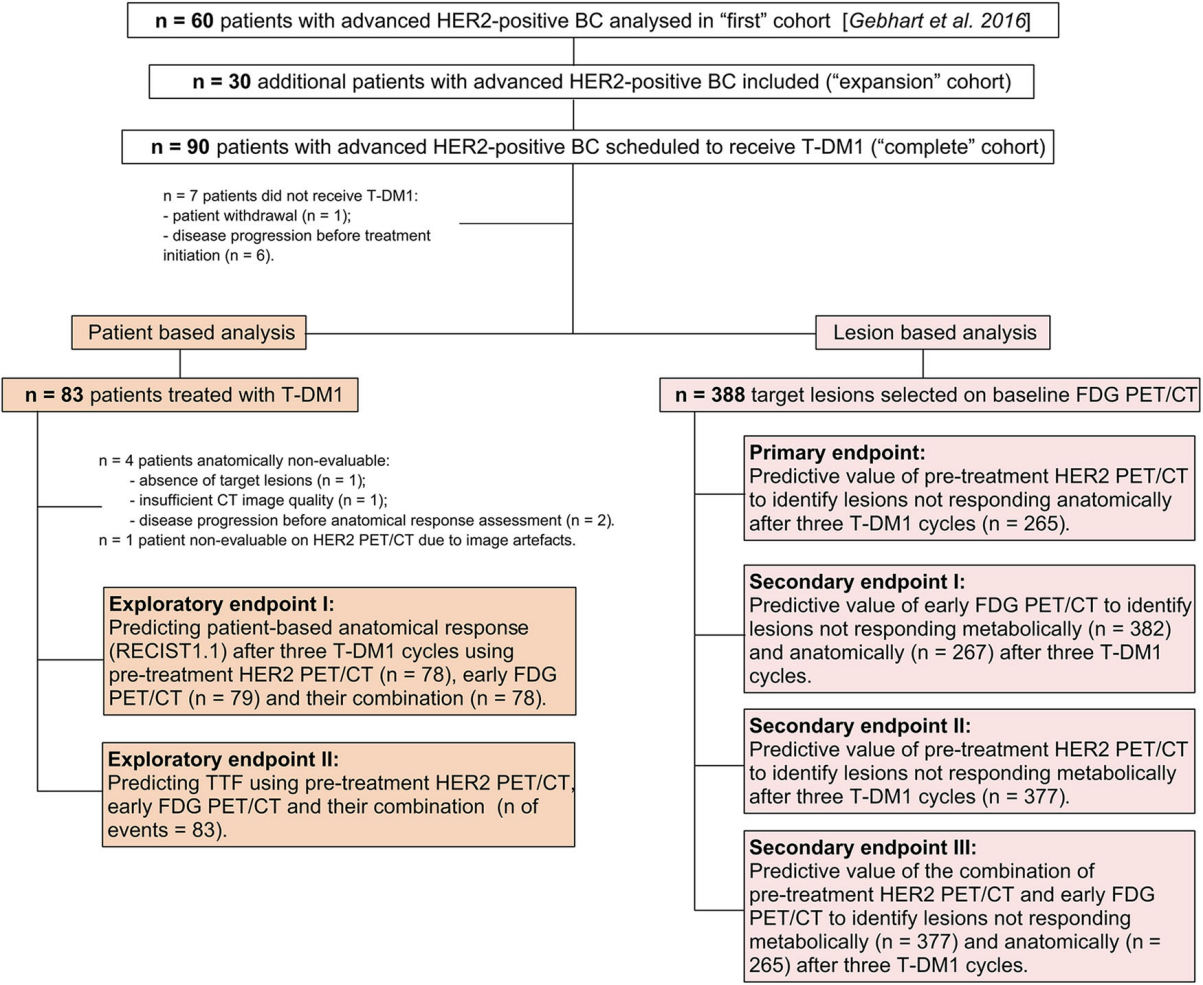
Study flow chart of patients and lesions included in the analysis of the primary, secondary and exploratory endpoints of ZEPHIR clinical trial. n number, BC breast cancer, T-DM1 trastuzumab emtansine, TTF time-to-treatment failure.

An exploratory objective of our study was a patient-based analysis in which the negative and positive predictive values (NPV and PPV) of HER2 PET/CT, early FDG response, and their combination are compared with RECIST 1.1 response evaluation after three T-DM1 cycles and correlated with time-to-treatment failure (TTF). Previously, we published the results of an interim patient-based analysis with the same objective, including only the first 60 patients enrolled in the study^14^. Here, we provide the updated results of the patient-based analysis in the “complete” study cohort of 90 patients, as well as the results of a subgroup analysis performed in the 30 additional patients which were included, i.e., the “expansion” cohort (Fig. 1).

Finally, with rapidly increasing healthcare expenditures due to an aging population, development of new technologies, and increasing personnel costs, we performed a simple cost estimation implicating the proposed PET/CT-guided management of patients receiving T-DM1 based on the accuracy data of the ZEPHIR study.

## Results

### Patients and treatment results of T-DM1

Between May 2012 and February 2017, 90 patients were included in the study and were scheduled to receive T-DM1 (Fig. 1). Patients’ characteristics are detailed in Table 1. Eighty-three patients received a median of 12 cycles of T-DM1 (range 1–114). Median TTF of the complete patient cohort was 7.7 months (95% CI: 6.4–9.6 months). At the time of analysis (May 2022), T-DM1 had been discontinued in all patients, with four still in follow-up receiving trastuzumab only (*n* = 2) or trastuzumab and hormonal therapy with letrozole (*n* = 2). Reasons for T-DM1 discontinuation were disease progression in 82% (68/83) and toxicity in 16% (13/83). T-DM1 was discontinued at the request of the patient or treating physician in the two remaining cases after receiving 46 and 114 T-DM1 cycles, respectively. Both patients experienced very good partial response and were still in follow-up at the time of the analysis. In an effort to better characterize such cases of long benefit from T-DM1, we identified 12 exceptional responders, i.e., the patients with TTF at least 3 times longer than the median TTF of the complete cohort^15^. They had minimum TTF of 25.8 months, number of prior treatments ranging from 0 to 6, and received a median of 65 T-DM1 cycles (range 32–114). Interestingly, all exceptional responders had ^89^Zr-trastuzumab image pattern A (*n* = 9) or B (*n* = 3), i.e., they were categorized as HER2-positive on the baseline HER2 PET/CT, and 10 out of 12 achieved partial RECIST1.1 response after the third T-DM1 cycle (*n* = 1 patient had stable disease and *n* = 1 patient was non-evaluable).

**Table 1:** Baseline patients’ characteristics.

Age at inclusion	
Median in years (Range)	54 (30–78)
ECOG - *n* patients	
0	46
1	44
Disease type at screening	
Visceral	84
Non-visceral	6
History of brain metastases - *n* patients	20
HER2-positivity based on primary tumor - *n* patients	84
Confirmed by reference lab	82
Not confirmed by reference lab	2
FISH+ only	4
IHC 2+ with FISH+	11
IHC 3+ with FISH+	42
IHC 3+ (no FISH+)	27
HER2-positivity based on metastatic biopsy - *n* patients	6
Confirmed by reference lab	6
Not confirmed by reference lab	0
FISH+ only	0
IHC 2+ with FISH+	3
IHC 3+ with FISH+	2
IHC 3+ (no FISH+)	1
Hormone receptor status - *n* patients	
ER+ or PR+ or both	63
ER- and PR-	27
Prior systemic therapies for advanced disease	
Yes - *n* patients	83
No - *n* patients	7
Median number of lines (range)	3 (0–11)
Median number of lines including trastuzumab (range)	2 (0–7)
Tyrosine kinase inhibitor - number of patients	24
Pertuzumab - *n* patients	6
HSP90 inhibitor - *n* patients	4

*n* number, *ICH* immunohistochemistry, *FISH* fluorescence in-situ hybridization, *ER* estrogen receptor, *PR* progesterone receptor, *HSP90* heat shock protein.

### Safety

No adverse events (AEs) related to HER2 PET-tracer injection were reported in the entire cohort. Grade ≥3 AEs due to T-DM1 were reported in 48 patients (53.3%), among which the most common were fatigue (11.1%), hypertension (7.8%), and increased gamma-glutamyl transferase (16.7%), leading to dose reductions in 20 patients. Thirty-three serious AEs were reported for 20 patients, with 11 possibly related to T-DM1, including thrombocytopenia (*n* = 2), pyrexia and chills (*n* = 4), anal abscess, pulmonary tumor hemorrhage, cognitive disorder, seizure and supraventricular tachycardia (all *n* = 1).

### Lesion-based molecular imaging results

Three hundred eighty-eight tumor lesions were selected on baseline FDG PET/CT. HER2 lesion-based classification was performed for 383, as 5 were excluded due to HER2 PET/CT image artifacts. The distribution among HER2 classes is presented in Supplementary Fig. 2. Over one-third of all lesions (39%) were categorized as HER2-negative: 84/383 (22%) in class 1, and 64/383 (17%) in class 2, reflecting considerable heterogeneity of HER2 overexpression as detected on HER2 targeted imaging.

The anatomic response was evaluable for 265/383 target lesions (measurable per RECIST1.0), of which 93 (35%) were HER2-negative. HER2 PET/CT correctly identified 75/93 HER2-negative lesions as anatomically NR after three T-DM1 cycles with an NPV of 81% (Table 2).

**Table 2:** Relation between HER2 classification of tumor lesions and anatomic response measurements.

	Classification	n lesions	Anatomic lesion response after three T-DM1 cycles		PPV	NPV
			R	NR		
HER2 PET/CT	+	172	123	49	72%^1^	
	−	93	18	75		81%^2^

Exact 95% confidence intervals as follows: ^1^: 64–78%, ^2^: 71–88%.

*n* number, *HER2+* HER2-positive lesions (class 3 and 4), *HER2-* HER2-negative lesions (class 1 and 2), *R* anatomically responding lesions, *NR* anatomically non-responding lesions.

Late metabolic response was evaluable in 377 out of 383 lesions (6 lesions excluded due to disease progression before late FDG PET/CT was performed). The PPV and NPV of HER2 PET/CT for the lesion-based late metabolic response were 86% and 63%, respectively (Supplementary Table 2A).

The predictive values of early FDG PET/CT response alone and in combination with the pre-treatment HER2 PET/CT in selecting non-responding lesions, according to anatomic and metabolic response criteria, are detailed in Supplementary Table 2B and 2C, respectively. Briefly, out of 109 lesions classified as mNR on the early FDG PET/CT, only 21 showed anatomic response on the diagnostic CT after three T-DM1 cycles, giving NPV of 81% for the early FDG PET/CT lesion-based evaluation. When combining both molecular imaging results, the NPV for the absence of anatomic response after three T-DM1 cycles was 91%. The combination of HER2 and early FDG PET/CT resulted in an NPV of 84% and PPV of 97% for the late metabolic response.

### Patient-based molecular imaging results

A complete summary of the relation between RECIST 1.1 response and results from HER2 PET/CT and FDG PET/CT in both the “complete” and the “expansion” cohort is presented in Table 3.

**Table 3:** Relation between patient RECIST 1.1 response and results of HER2 PET/CT, early FDG PET/CT and their combination.

A	Patient-based analysis of the “complete” cohort (*n* = 90)					
	Classification	n patients	RECIST 1.1 after three T-DM1 cycles		PPV	NPV
			R	NR		
HER2 PET/CT	+	53	41	12	77%^1^	
	−	25	4	21		84%^2^
Early FDG PET/CT	R	42	40	2	95%^3^	
	NR	37	6	31		84%^4^
HER2 PET/CT/Early FDG PET/CT	+/R	36	36	0	100%	
	+/NR	17	5	12		
	−/R	6	4	2		
	−/NR	19	0	19		100%
B	Subgroup patient-based analysis of the “expansion” cohort (*n* = 30)					
	Classification	*n* patients	RECIST 1.1 after three T-DM1 cycles		PPV	NPV
			R	NR		
HER2 PET/CT	+	14	13	1	93%^1^	
	−	9	2	7		78%^2^
Early FDG PET/CT	R	15	14	1	93%^3^	
	NR	8	1	7		88%^4^
HER2 PET/CT/Early FDG PET/CT	+/R	12	12	0	100%	
	+/NR	2	1	1		
	−/R	3	2	1		
	−/NR	6	0	6		100%

Exact 95% confidence intervals as follows:^1^: 64–88%,^2^: 64–95%, ^3^: 84–99%, ^4^: 68–94%.

Exact 95% confidence intervals as follows: ^1^: 66–100%, ^2^: 40–97%, ^3^: 68–100%, ^4^: 47–100%.

*n* number, *HER2+* HER2-positive patients, *HER2-* HER2-negative patients, *R* responders, *NR* non-responders.

In the “complete” patient cohort, HER2 PET/CT and early FDG PET/CT predicted the absence of RECIST 1.1 response after three T-DM1 cycles, each with an NPV of 84%. When combining the results of both molecular imaging modalities, all of the HER2-negative patients and early metabolic non-responders were classified as non-responders according to RECIST 1.1 (NPV 100%). Discordance between the HER2-positivity and early metabolic response in terms of HER2-positive/early metabolic non-responders and HER2-negative/early metabolic responders was found in 23 patients. HER2 PET/CT correctly predicted response in 30% of the discordant patients, whereas early FDG PET/CT was correct in 70% of the cases.

For the patient-based analysis of the “expansion” cohort, RECIST 1.1 response was available in 23 out of 30 patients, as five received no T-DM1 due to progression before treatment initiation, and in two patients, clinical progression occurred before administration of the third T-DM1 cycle, and thus no CT for anatomic response assessment was performed. HER2 PET/CT and early FDG PET/CT predicted the absence of RECIST1.1 response after three T-DM1 cycles with NPVs of 78% and 88%, respectively. The combination of both imaging modalities correctly identified all patients who did or did not show objective RECIST 1.1 response after three T-DM1 cycles with PPV and NPV of 100%.

### Time to treatment failure

**Fig. 2:**
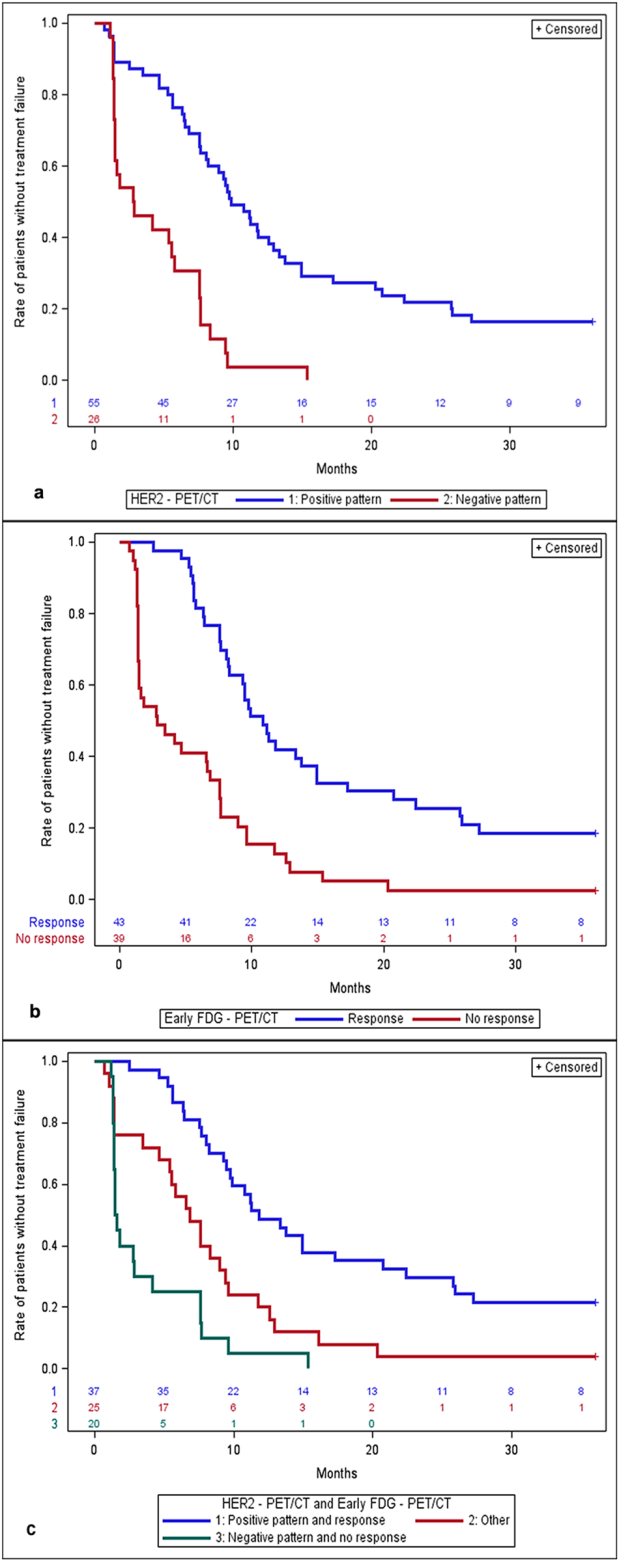
Time-to-treatment failure according to HER2 PET/CT alone, early FDG PET/CT alone, and a combination of the HER2 PET/CT and early FDG PET/CT. **a** Early FDG PET/CT alone (**b**) combination of the HER2 PET/CT and early FDG PET/CT (**c**). **a** HER2-positive pattern (blue line): patients with pattern A and B for HER2 uptake; HER2-negative pattern (red line): patients with pattern C and D for HER2 uptake. **b** Patients with early metabolic response on FDG PET/CT (response, blue line); patients without early metabolic response on FDG PET/CT (no response, red line). **c** Patients with positive pattern on HER2 PET/CT showing early metabolic response on FDG PET/CT (blue line); patients with negative pattern on HER2 PET/CT without early metabolic response on FDG PET/CT (green line); patients with positive pattern on HER2 PET/CT and early nonresponse or vice versa (discordant cases) (red line).

The molecular imaging results of all patients receiving T-DM1 (*n* = 83) were correlated with treatment discontinuation (Fig. 2). Median TTF was 9.9 months (95% CI: 7.7–12.9 months) for the HER2-positive patients versus 2.8 months (95% CI: 1.4–5.8 months) in the HER2-negative group of patients, with an HR of 3.7 (95% CI: 2.19–6.35, *p* < 0.0001) using the HER2-positive group as reference. According to the early metabolic response assessment, the median TTF was 10.8 months in early metabolic responders (95% CI: 8.2 months – 14.9) and 2.8 months (95% CI: 1.4–6.8 months) for the early metabolic non-responders (HR of 3.0, 95% CI: 1.9–4.8, *p* < 0.0001). When combining both imaging modalities, HER2-positive and early metabolic responding patients had a median TTF of 11.8 months (95% CI: 9.5–20.8 months), compared to the HER2-negative and early metabolic non-responding patients who had a median TTF of 1.5 months (95% CI: 1.4–4.2 months). Patients with discordance between HER2-positivity and the early metabolic response (as defined above) demonstrated a median TTF of 6.8 months (95% CI: 4.7–9.4 months). Using the double-positive group as a reference, the HRs for the double-negative and discordant groups were 5.8 (95% CI: 3.1–10.8) and 2.6 (95% CI: 1.5–4.5), respectively (overall *p* < 0.0001).

### Cost-effectiveness of imaging in the ZEPHIR trial

**Fig. 3:**
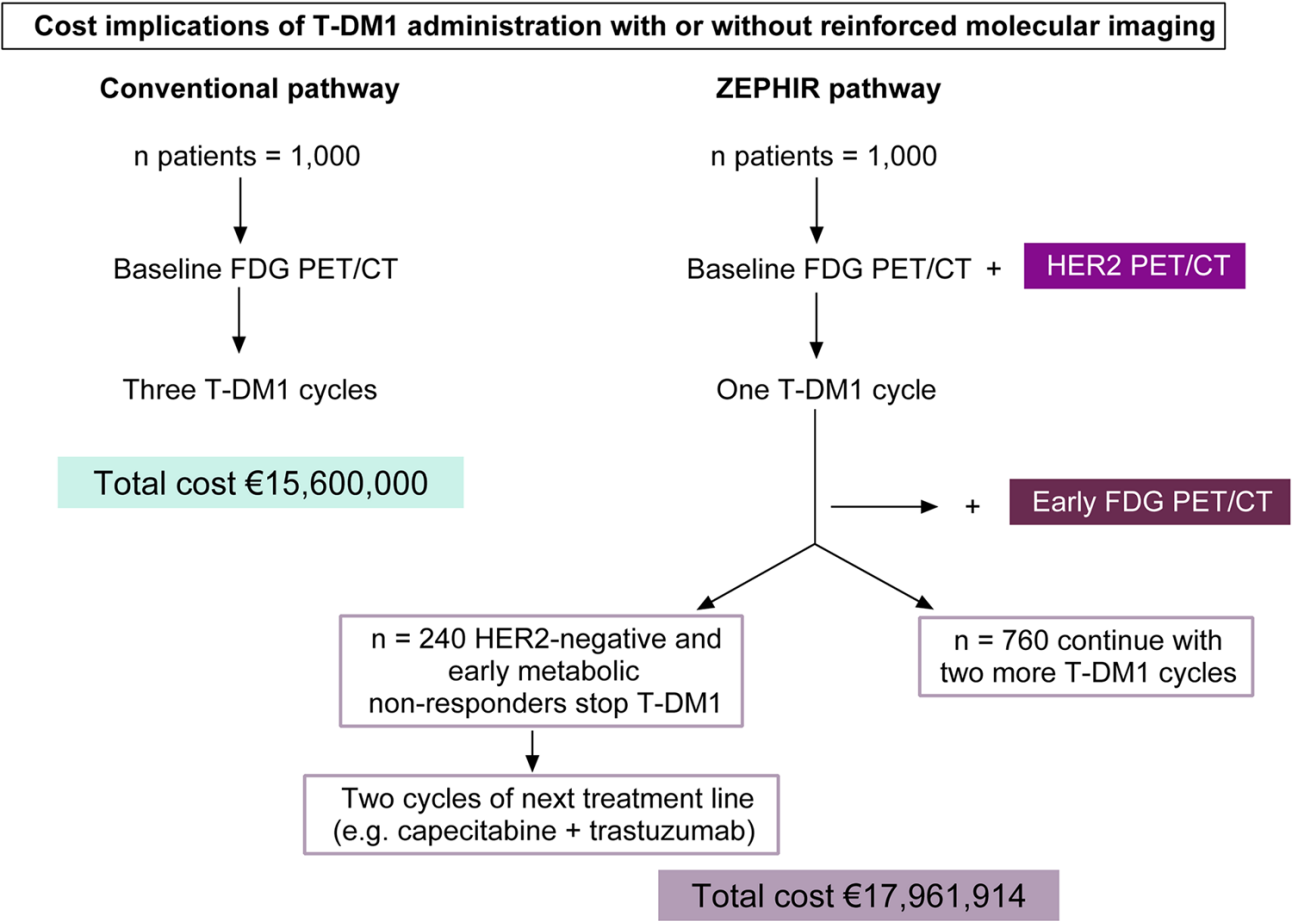
Cost estimation of two hypothetical cohorts of 1000 patients each with HER2-positive breast cancer, entering a conventional pathway and the ZEPHIR pathway. n number, T-DM1 trastuzumab emtansine.

In the conventional pathway, as presented in Fig. 3, all 1000 patients would receive three T-DM1 cycles. The cost of the treatment and outpatient hospitalization in this group is estimated at €15,600,000. In the ZEPHIR pathway, all 1000 patients would undergo baseline HER2 PET/CT, one T-DM1 cycle given in the outpatient department of the hospital, and an early FDG PET/CT at a cost of €8824,300. As the baseline FDG PET/CT would be performed identically in both pathways, its cost was excluded from the analysis. Given the NPV for RECIST1.1 response of the combination of both imaging modalities of 100%, T-DM1 would be ceased after one cycle in 240 HER2-negative and early metabolically non-responding patients. Instead, they would receive a next treatment line with capecitabine + trastuzumab at an additional cost of €1,233,614 for two cycles. The other 760 patients would continue with two more T-DM1 administrations at the cost of €7904,000. Thus, the total cost of the ZEPHIR pathway is estimated at €17,961,914, which results in an incremental cost of €2361.9 per patient compared to the conventional pathway. The one and a half months (i.e., two T-DM1 administrations) gained in the 240 patients creates an incremental cost-effectiveness ratio (ICER) of €18,895.2 for the ZEPHIR pathway.

In a “HER2-only imaging” ZEPHIR pathway (excluding the FDG PET/CT scans), 320 of the 1000 patients would not start T-DM1, based on the percentage of HER2-negative patients in the “complete” ZEPHIR cohort. The €4,992,000 for three T-DM1 cycles (administered during outpatient care), saved in these 320 patients, is compared to the cost of performing HER2 PET/CT in all 1000 patients, resulting in an overall cost saving of €1,992,000.

## Discussion

Our study prospectively evaluated PET imaging as a powerful tool for better treatment individualization and early prediction of T-DM1 response in patients with advanced HER2- positive breast cancer. Up to now, not a single predictive biomarker of T-DM1 efficacy in HER2- positive breast cancer has been identified: HER2 mRNA expression and mutations of the *PiK*_*3*_*CA* gene (encoding the p110α catalytic subunit of the phosphatidylinositol 3-kinase), for example, turned out to be “prognostic” but not “predictive” biomarkers in the advanced disease trials^16^. This is a limitation in the era of personalized oncology and also given the relatively high cost of the drug.

Being the first prospective and comprehensive imaging study in advanced HER2-positive breast cancer, we could not formulate a patient-oriented statistical hypothesis, which is why the primary endpoint of our study was a lesion-based analysis. In the complete patient cohort, a considerable number of lesions (39%) did not show sufficient target expression on HER2 PET/CT. Moreover, these lesions were less likely to respond anatomically after three cycles of T-DM1 than lesions showing high ^89^Zr-trastuzumab uptake on PET imaging. Further investigation is required to understand why in 18 out of 93 HER2-negative lesions on HER2 PET/CT, an objective anatomic response was still observed after three cycles of T-DM1. Our study could not distinguish between a real lack of receptor overexpression, receptor masking^17,18^, or induced response in low HER2 availability due to a high potency of DM1 in the absence of intracellular resistance mechanisms. In “HER2-low” tumors (defined as an ICH score of 1+ or 2+ and negative results on FISH), a new generation of ADC, trastuzumab deruxtecan, prolongs progression-free survival and overall survival compared to the physician’s choice of chemotherapy^19^. Due to its highly potent and membrane-permeable cytotoxic payload, along with its enzyme-cleavable antibody–drug linker and high drug-to-antibody ratio, trastuzumab deruxtecan has antitumor activity in breast cancers with low levels of HER2 through the bystander effect^20^. A similar mechanism could explain the response to T-DM1 we observed in our study’s lesions classified as HER2-negative on the pre-treatment HER2 PET/CT. By adding the early metabolic response assessment, only 6 out of 65 HER2-negative and early mNR lesions were falsely predicted as anatomically NR, reaching a clinically relevant NPV of 91%. We are currently performing DNA/RNA sequencing of a selected number of biopsied metastatic lesions to explain potential differences in tracer uptake and potential contribution of ctDNA monitoring to improve response prediction early during treatment. Nevertheless, patients included in the ZEPHIR trial had received multiple treatment lines, potentially influencing an overall receptor status change and increasing disease heterogeneity. The clinical utility of HER2 PET/CT and early response measurement on FDG PET/CT is being evaluated in the setting of first presentation of metastatic breast cancer as part of an ongoing prospective trial (IMPACT trial, clinicaltrial.gov identifier: NCT01957332)^21^. Awaiting results of this study will potentiate the role of molecular imaging within the framework of standardized assessments, especially in the early disease stages and first-line systemic therapy.

In the previously published interim analysis of the first 60 patients included in our study, we demonstrated clinically relevant NPVs for HER2 PET/CT (88%) and early FDG PET/CT (83%) in terms of RECIST 1.1 patient response. By combining both imaging modalities, we accurately identified those patients who will not benefit from T-DM1 with an NPV for RECIST 1.1 response of 100%^14^. These remarkable results are further supported in the current analysis of the “complete” cohort of 90 patients and, more importantly, are validated by the findings of the analysis we performed in the “expansion” cohort. Indeed, combining results from both imaging modalities again showed clear discrimination of responders versus non-responders with a PPV and NPV of 100%. Approximately one-third of the patients again had little or no ^89^Zr-trastuzumab uptake across their metastases and experienced a shorter TTF than patients classified as HER2-positive. In discordant cases (HER2-positive patients but early metabolic non-responders or vice versa), FDG PET/CT was more accurate than the HER2 PET/CT in predicting RECIST 1.1 response. This contribution of early metabolic response assessment to the pre-treatment HER2 PET/CT can be partly attributed to non-HER2 related mechanisms of a resistance to T-DM1 (even in the presence of the HER2 expression as a prerequisite for its activity). Namely, several resistance mechanisms to T-DM1 have been proposed, including changes in the intracellular concentrations of the T-DM1 payload, altered expression of drug efflux transporters, and resistance to the cytotoxic effect of the released tubulin inhibitor payload^22^. Understanding these mechanisms is important for optimizing the development of new HER2-targeted ADCs.

Following our study objective on identifying early on lesions and patients not responding to the treatment, and substantiated by a previous work from our group^12^, we opted to use a relatively low threshold for the early metabolic response criteria (15% after one treatment cycle). In addition, in order to avoid unjustified discontinuation of potentially beneficial therapy, it’s essential for early non-response detection to have a strong NPV, achieved through the use of minimal response threshold.

Despite the incremental cost of €2361.9 per patient, we demonstrated in our cost-effectiveness analysis that we create extra value for the patients in the ZEPHIR pathway compared to the conventional pathway by early terminating an ineffective treatment with potential toxicity. The ICER of €18,895.2 for the ZEPHIR pathway is lower than some of the thresholds commonly used in cost-effectiveness analyses. For example, in a recent study evaluating the cost-effectiveness of treatment of oligometastatic prostate cancer in Belgium, the willingness-to-pay threshold was set at €40,000^23^. The Institute for Clinical and Economic Review in the United States justifies an ICER of up to $150,000 when conducting drug value-based analyses^24^. Based on this simplified model, the ZEPHIR pathway is estimated to be cost-effective when applied in clinical practice in patients with breast cancer scheduled to receive T-DM1. Alternatively, if the decision to initiate T-DM1 is guided solely by the baseline HER2 PET/CT, a cost saving of €1992 per patient would be achieved. This decision should ideally be taken in consultation with the patient, bearing in mind the small portion of HER2-negative patients responding anatomically after three T-DM1 cycles (16%).

We assessed target lesions on HER2 PET/CT only qualitatively for the presence or absence of uptake compared to the healthy tissue without performing a semi-quantitative analysis using SUV, which might be considered as a limitation of our study. However, further inter-institutional optimization of HER2 PET acquisition and reconstruction parameters is needed to rely on SUV cutoffs to classify HER2-positivity correctly. By performing a semi-quantitative analysis of ^89^Zr-trastuzumab uptake measured as SUVmax, one group showed a higher SUVmax in patients with HER2-positive disease versus HER2-negative disease; however, only when hepatic metastases were excluded from the analysis^25^.

The relatively small number of patients analyzed in the “expansion” cohort is another limitation of our study. However, a true “validation” set would have required the inclusion of 300 patients, which was beyond the scope of the ZEPHIR trial.

In conclusion, we showed that molecular imaging, with its noninvasiveness and ability to evaluate the entire disease burden, can assess HER2 heterogeneity in HER2-positive breast cancer and can predict very early on lesions and patients not responding to the “parent” antibody-drug conjugate T-DM1. We believe similar efforts should be displayed for improved tailoring of the highly active but also toxic new generations of antibody-drug conjugates.

## Methods

### Key eligibility criteria and study design

The ZEPHIR study (ClinicalTrials.gov identifier: NCT01565200), is an international, single-arm phase II imaging trial conducted across five university hospitals in Belgium and The Netherlands, exploring the value of a pre-treatment HER2 PET/CT imaging in identifying tumor lesions (primary endpoint) and patients (exploratory endpoint) unlikely to respond to T-DM1 (Fig. 1). Patients with locally advanced or metastatic HER2-positive breast cancer were eligible for inclusion, with HER2 positivity of primary tumor or (recent) metastasis defined as FISH > 2.2 at the trial sites in Belgium and IHC3+ or IHC 2+ and FISH > 2.2 in The Netherlands. Other inclusion criteria are detailed in Supplementary Table 1. All patients underwent baseline whole-body contrast-enhanced diagnostic CT, or magnetic resonance imaging (MRI) in case CT contrast agent injection was contraindicated. Baseline HER2 and FDG PET/CT were performed maximum of seven days before treatment initiation. T-DM1 (3.6 mg/kg) was administered intravenously every three weeks (21 ± 3 days) according to standard clinical practice. Within the week preceding the second treatment cycle, an early metabolic response assessment with FDG PET/CT was done, the results of which, along with those of the baseline HER2 PET/CT, were blinded to the treating oncologist. After three T-DM1 cycles, diagnostic CT (or MRI) was performed for anatomic response assessment, and FDG PET/CT was performed for late metabolic response assessment. An overall response assessment (beyond cycle 3) was not performed because, after the third cycle of T-DM1, patients were followed at the discretion of the oncologists, according to routine clinical practice at each trial site. Treatment was continued until disease progression, unacceptable toxicity, or patient withdrawal from the study. In the event of toxicity, a dose delay or dose reduction to 3 or 2.4 mg/kg was allowed. Adverse events were graded and coded according to the National Cancer Institute Common Terminology Criteria for Adverse Events V4.0 and Medical Dictionary for Regulatory Activities (MedDRA). The study protocol was approved by the Medical Ethics Committee of the Institut Jules Bordet in Belgium and the Medical Ethical Review Committee of the University Medical Center Groningen in The Netherlands. All patients gave written informed consent. This study complied with all relevant ethical regulations, including the Declaration of Helsinki.

### Imaging procedures

Procedure guidelines based on European Association of Nuclear Medicine (EANM) recommendations for tumor PET imaging were used for performing FDG PET/CT. All participating centers were accredited under EARL (EANM Research Ltd.) accreditation program, allowing for the standardization of image quality^26^. Maximum standardized uptake value (SUVmax) corrected for lean body mass was used for tumor uptake quantification. HER2 PET/CT was acquired four days after injection of 37 MBq ± 10% ^89^Zr-trastuzumab and 50 mg of cold trastuzumab. Tracer preparation and image acquisition/reconstruction standardization were performed as reported earlier^27^. Image quality for molecular and anatomic imaging and compliance with imaging guidelines were centrally assessed by an imaging core laboratory (Orilab, Institut Jules Bordet).

### Selection of target lesions and lesion-based HER2 classification

Target lesions were first selected on baseline FDG PET/CT according to the following criteria defined in the study protocol: maximum of 10 (5 per organ) unequivocally neoplastic lesions per patient, with a size of ≥15 mm in axial diameter (≥10 mm in case of lymph node lesions), outside of any previous radiation field, and with SUVmax ≥ 1.5 × liver SUVmean + 2 standard deviations (SD) in 3-cm-diameter spherical region-of-interest (ROI) in normal liver (or SUVmax > 2.0 × blood SUVmean + 3 SD in 1-cm-diameter ROI in descending thoracic aorta if the liver is abnormal), were eligible for target lesion selection.

**Fig. 4:**
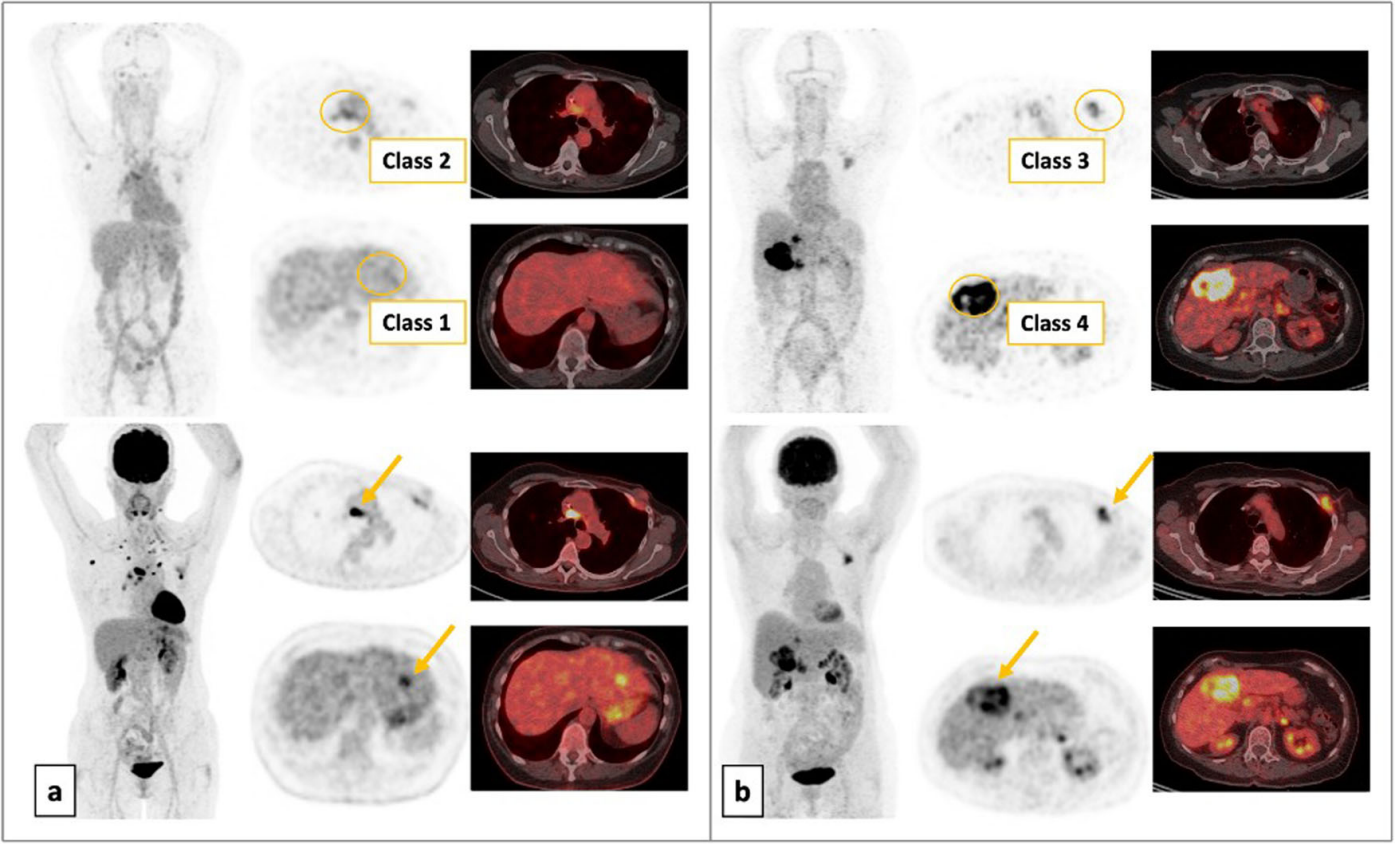
Patterns and classification of ^89^Zr-trastuzumab lesion uptake based on visual assessment, confronted with an FDG PET/CT. **a** HER2 PET/CT (upper row, maximum intensity projection (MIP) and axial PET and fusion images) in a patient with liver lesion showing no ^89^Zr-trastuzumab uptake (class 1, HER2-negative lesion) and a mediastinal lymph node showing uptake close to background activity (class 2, HER2-negative lesion) confronted with the FDG PET/CT images (lower row, MIP and axial PET and fusion images). **b** HER2 PET/CT (upper row, MIP and axial PET and fusion images) in a patient with axillary lymph node showing visible ^89^Zr-trastuzumab uptake (class 3, HER2-positive lesion) and a liver lesion showing clearly visible, high uptake (class 4, HER2-positive lesion) confronted with the FDG PET/CT images (lower row, MIP, and axial PET and fusion images).

Based on the ^89^Zr-trastuzumab uptake, a visual analysis of the pre-treatment HER2 PET/CT categorized the target lesions into “class 1” (same as surrounding background activity), “class 2” (visible but close to surrounding background activity), “class 3” (visible and differentiable from surrounding background activity), and “class 4” (clearly visible, high uptake). Classes 1 and 2 were considered HER2-negative lesions, and 3 and 4 as HER2-positive lesions (Fig. 4).

### Lesion-based response assessment

Those target lesions, measurable per RECIST 1.0, were followed on diagnostic CT or MRI and were eligible for anatomic response assessment. The measurability of lesions was determined according to RECIST 1.0, which allows lymph nodes with a diameter of ≥ 10 mm to be included in the lesion-based response anatomic response assessment^28^. Anatomic lesion response was defined as a decrease in axial diameter of more than 30% from baseline. Lesions showing ≥20% increase in size from baseline imaging were considered progressive. Anatomically stable (<30% size decrease) and progressive lesions were considered non-responding lesions (NR), and those with partial or complete anatomic response were considered responding lesions (R).

Metabolic response assessment of the selected target lesions was done on the early FDG PET/CT and on the late FDG PET/CT performed after three cycles of T-DM1. Metabolic lesion response was based on EORTC (European Organization for Research and Treatment of Cancer) criteria for the early assessment, with lesion response cutoff at a minimum 15% decrease in SUVmax^29^. For the late metabolic response assessment, a cutoff of a minimum 30% decrease in SUVmax was used^30^. Metabolic lesion progression in both cases was considered as >25% SUVmax increase from baseline. Lesions not fulfilling the response criteria, i.e., metabolically stable (<15% and <30% SUVmax decrease for early and late FDG PET/CT, respectively) and metabolically progressive lesions were considered metabolically non-responding (mNR) lesions, as opposed to lesions showing partial or complete metabolic response which were considered metabolically responding lesions.

### Patient-based HER2 classification

At the patient level, a side-by-side comparison of the baseline FDG PET/CT and HER2 PET/CT was made, and four HER2 image patterns were identified according to the proportion of FDG avid tumor load showing relevant ^89^Zr-trastuzumab uptake: A (entire tumor load showed pertinent tracer uptake), B (dominant part of tumor load showed tracer uptake), C (minor part of tumor load showed tracer uptake) and D (entire tumor load lacked tracer uptake) as shown in Supplementary Fig. 1. Unlike the lesion-based HER2 classification, which was performed on individual target lesions, the patient-based HER2 classification considered the entire tumor load as shown on the FDG PET/CT and the aforementioned image patterns were assigned based on the overall presentation of the tumor. Patients with patterns A and B were considered HER2-positive, and those with patterns C and D were HER2-negative.

### Patient-based response assessment

The patient-based anatomic response was assessed using RECIST 1.1^31^. A maximum of 5 target lesions (maximum 2 per organ), measurable according to RECIST 1.1, were selected from baseline diagnostic CT or MRI (in *n* = 2 patients). The response assessment was performed after three cycles of T-DM1. Patients showing a complete or partial response were classified as responders, whereas patients with stable or progressive disease were considered non-responders.

The patient-based metabolic response was assessed on the early FDG PET/CT performed after only one T-DM1 cycle. Based on the metabolic imaging, patients were classified into four groups: “class I” (all lesions show a significant metabolic response: minimum 15% decrease in SUVmax), “class II” (mixed response with the dominant response, i.e., more than 50% of lesions show a significant metabolic response), “class III” (mixed response, dominant nonresponse), and “class IV” (no lesion is responding, or presence of at least one metabolically progressive or new lesion). Patients in classes I and II were considered early metabolic responders, and those in classes III and IV were early metabolic non-responders.

The analysis of the “expansion” cohort, i.e., the patient-based HER2 classification and response assessment (anatomic response as per RECIST1.1 and metabolic response on the early FDG PET/CT) for the 30 patients additionally included after the “first” cohort, were performed in the same manner as described above.

Two independent nuclear medicine physicians reviewed all PET images, after which discordances were revised, and a consensus was reached. A senior radiologist centrally reviewed diagnostic CT/MRI images.

### Cost estimation data

Cost estimation was performed on two hypothetical cohorts of 1000 patients with HER2-positive locally advanced or metastatic breast cancer starting a T-DM1 treatment, one group entering a conventional pathway and the other group entering the ZEPHIR pathway, detailed below. The accuracy data and relative costs (based on reimbursement prices of the Belgian National Institute for Health and Disability Insurance) used in the analysis are displayed in Supplementary Table 3.

### Statistical analysis

The study’s underlying hypothesis was that a lesion with negative uptake on baseline HER2 PET/CT would not respond anatomically to T-DM1. Using a single-stage Fleming-A’Hern design with a one-sided test (5% type I error) and power of 80% to test the null hypothesis of an NPV of <85% versus the alternative NPV of ≥95%, we calculated that 60 HER2-negative and RECIST 1.0 measurable lesions would need to be examined. The secondary objectives aimed to show that HER2 PET/CT, early FDG PET/CT, and the combination of both HER2 PET/CT and early FDG PET/CT would be able to select lesions not responding to treatment according to metabolic and anatomic response criteria post three cycles of T-DM1. To get a power of 90% with a one-sided test (5% type I error) for testing the null hypothesis of NPV < 85% versus the alternative hypothesis of NPV ≥ 95%, we calculated that 76 mNR FDG-positive lesions on the early FDG PET/CT would need to be examined. The NPVs for both primary and secondary objectives were calculated and reported together with an associated exact 95% confidence interval (CI). Finally, for the exploratory objective of our study, we defined TTF as the time from the start of T-DM1 to discontinuation for any reason, including disease progression (clinical or image-based), treatment toxicity, and death. For the correlation between TTF and imaging results, patients who discontinued T-DM1 for any other reason than progression were censored. The distribution of TTF was estimated by the Kaplan-Meier method. For comparison, the data were fitted with Cox regression models. Hazard ratios (HRs) are reported with 95% CI. The statistical significance level was set at 5%.

### Supplementary information

Supplemental material

Related Manuscript File

## Data Availability

All the data that support the findings in this study are available from the corresponding author upon reasonable request.

## References

[1] Bray F (2018). Global cancer statistics 2018: GLOBOCAN estimates of incidence and mortality worldwide for 36 cancers in 185 countries. CA Cancer J. Clin..

[2] Loibl S, Gianni L (2017). HER2-positive breast cancer. Lancet.

[3] Junttila TT, Li G, Parsons K, Phillips GL, Sliwkowski MX (2011). Trastuzumab-DM1 (T-DM1) retains all the mechanisms of action of trastuzumab and efficiently inhibits growth of lapatinib insensitive breast cancer. Breast Cancer Res. Treat..

[4] Verma S (2012). Trastuzumab emtansine for HER2-positive advanced breast cancer. N. Engl. J. Med..

[5] von Minckwitz G (2019). Trastuzumab emtansine for residual invasive HER2-positive breast cancer. N. Engl. J. Med..

[6] Wolff AC (2018). Human epidermal growth factor receptor 2 testing in breast cancer: American Society of Clinical Oncology/College of American Pathologists Clinical Practice Guideline focused update. Arch. Pathol. Lab. Med..

[7] Seol H (2012). Intratumoral heterogeneity of HER2 gene amplification in breast cancer: its clinicopathological significance. Mod. Pathol..

[8] Miglietta F (2021). Evolution of HER2-low expression from primary to recurrent breast cancer. NPJ Breast Cancer.

[9] Gennari A (2021). ESMO clinical practice guideline for the diagnosis, staging and treatment of patients with metastatic breast cancer. Ann. Oncol..

[10] Dijkers EC (2010). Biodistribution of 89Zr-trastuzumab and PET imaging of HER2-positive lesions in patients with metastatic breast cancer. Clin. Pharm. Ther..

[11] Bensch F (2018). (89)Zr-trastuzumab PET supports clinical decision making in breast cancer patients, when HER2 status cannot be determined by standard work up. Eur. J. Nucl. Med. Mol. Imaging.

[12] Gebhart G (2013). 18F-FDG PET/CT for early prediction of response to neoadjuvant lapatinib, trastuzumab, and their combination in HER2-positive breast cancer: results from Neo-ALTTO. J. Nucl. Med..

[13] Perez-Garcia JM (2021). Chemotherapy de-escalation using an (18)F-FDG-PET-based pathological response-adapted strategy in patients with HER2-positive early breast cancer (PHERGain): a multicentre, randomised, open-label, non-comparative, phase 2 trial. Lancet Oncol..

[14] Gebhart G (2016). Molecular imaging as a tool to investigate heterogeneity of advanced HER2-positive breast cancer and to predict patient outcome under trastuzumab emtansine (T-DM1): the ZEPHIR trial. Ann. Oncol..

[15] Mullard A (2021). Parsing exceptional responders. Nat. Rev. Drug Discov..

[16] Perez EA (2019). Relationship between tumor biomarkers and efficacy in MARIANNE, a phase III study of trastuzumab emtansine +/- pertuzumab versus trastuzumab plus taxane in HER2-positive advanced breast cancer. BMC Cancer.

[17] Wimana Z (2015). Mucolytic agents can enhance HER2 receptor accessibility for [(89)Zr]trastuzumab, improving HER2 imaging in a mucin-overexpressing breast cancer xenograft mouse model. Mol. Imaging Biol..

[18] Palyi-Krekk Z (2007). Hyaluronan-induced masking of ErbB2 and CD44-enhanced trastuzumab internalisation in trastuzumab resistant breast cancer. Eur. J. Cancer.

[19] Modi S (2022). Trastuzumab deruxtecan in previously treated HER2-low advanced breast cancer. N. Engl. J. Med..

[20] Ogitani Y, Hagihara K, Oitate M, Naito H, Agatsuma T (2016). Bystander killing effect of DS-8201a, a novel anti-human epidermal growth factor receptor 2 antibody-drug conjugate, in tumors with human epidermal growth factor receptor 2 heterogeneity. Cancer Sci..

[21] Imaging patients for cancer drug selection—metastatic breast cancer (IMPACT-MBC). ClinicalTrials.gov identifier: NCT01957332. Updated January 5, 2022. Accessed 27 April 2023 (https://ClinicalTrials.gov/show/NCT01957332).

[22] Hunter FW (2020). Mechanisms of resistance to trastuzumab emtansine (T-DM1) in HER2-positive breast cancer. Br. J. Cancer.

[23] De Bleser E (2020). A trial-based cost-utility analysis of metastasis-directed therapy for oligorecurrent prostate cancer. Cancers.

[24] Sussman M, Yu JC, Menzin J (2020). Do research groups align on an intervention’s value? Concordance of cost-effectiveness findings between the institute for clinical and economic review and other health system stakeholders. Appl. Health Econ. Health Policy.

[25] Dehdashti F (2018). Evaluation of [(89)Zr]trastuzumab-PET/CT in differentiating HER2-positive from HER2-negative breast cancer. Breast Cancer Res. Treat..

[26] Boellaard R (2015). FDG PET/CT: EANM procedure guidelines for tumour imaging: version 2.0. Eur. J. Nucl. Med. Mol. Imaging.

[27] Makris NE (2014). Multicenter harmonization of 89Zr PET/CT performance. J. Nucl. Med..

[28] Therasse P (2000). New guidelines to evaluate the response to treatment in solid tumors. European Organization for Research and Treatment of Cancer, National Cancer Institute of the United States, National Cancer Institute of Canada. J. Natl Cancer Inst..

[29] Young H, European Organization for Research and Treatment of Cancer (EORTC) PET Study Group (1999). Measurement of clinical and subclinical tumour response using [18F]-fluorodeoxyglucose and positron emission tomography: review and 1999 EORTC recommendations.. Eur. J. Cancer.

[30] Wahl RL, Jacene H, Kasamon Y, Lodge MA (2009). From RECIST to PERCIST: evolving considerations for PET response criteria in solid tumors. J. Nucl. Med..

[31] Eisenhauer EA (2009). New response evaluation criteria in solid tumours: revised RECIST guideline (version 1.1). Eur. J. Cancer.

