# Supplementary material for: Molecular imaging predicts lack of T-DM1 response in advanced HER2-positive breast cancer (final results of ZEPHIR trial)
Source: NPJ Breast Cancer. 2024 Jan 6;10:4. doi: 10.1038/s41523-023-00610-6 (PMC10771456; doi:10.1038/s41523-023-00610-6)
Supplement: Supplementary file 1 — Supplemental material [file 41523_2023_610_MOESM1_ESM.pdf]

**Supplementary Table 1.** Patients' inclusion and exclusion criteria	
Inclusion criteria	Exclusion criteria
1. The patient must have histologically confirmed HER2 positive invasive carcinoma of the breast in the reference laboratory of the participating center. HER2 positive criteria to be applied are those used in the participating countries:<br>- Belgium: FISH amplification ratio ≥ 2 in the reference laboratory of the participating center.<br>- The Netherlands: IHC 3+ or FISH ratio ≥ 2 in the reference laboratory of the participating center.<br>2. The patient must have documented progressive disease and present with at least 2 non-bone "target" metastatic lesions, unequivocally of neoplastic origin with a transaxial diameter greater than 2 cm on the screening diagnostic CT/MRI for all non-bone lesions except lymph nodes (a short axis greater than 1,5 cm for lymph nodes on the screening diagnostic CT/MRI). These two lesions should not be confluent with adjacent lesions and not have been irradiated previously.<br>3. A concurrent biopsy of a metastatic site is mandatory (with two formalin fixed paraffin-embedded (FFPE) core sample and two snap frozen tumor samples) after progression has been documented and before inclusion and the patient agrees with the procedure. Primary tumor blocks (or 11 unstained slides) available for confirmatory central laboratory HER2 testing in Institut Jules Bordet. If available, a snap-frozen sample of the primary tumor will also be centralized in Institut Jules Bordet.<br>4. Age ≥ 18 years.<br>5. Eastern Cooperative Oncology Group (ECOG) performance status (PS) 0 to 1.<br>6. No significant cardiac history and current LVEF ≥ 50%.<br>7. Adequate organ function, evidenced by the following laboratory results:<br>- Absolute neutrophil count > 1,500 cells/mm^3<br>- Platelet count > 100,000 cells/mm^3<br>- Hemoglobin > 9 g/dL<br>- AST(SGOT) and ALT (SGPT) < 2.5 x ULN<br>- Total Bilirubin ≤ 1.5 x ULN unless the patient has documented Gilbert's syndrome. Patients with known Gilbert's Syndrome should have direct bilirubin within normal limits.<br>- Serum alkaline phosphatase ≤ 2.5 x ULN. Patients with bone metastases: alkaline phosphatase ≤ 5 x ULN<br>- Serum creatinine < 2.0 mg/dL or 177 µmol/L<br>- International normalized ratio (INR) and activated partial thromboplastin time or partial thromboplastin time (aPTT or PTT) < 1.5 x ULN (unless on therapeutic anti-coagulation except vitamin K antagonists, which are prohibited in this study)<br>- Absence of any psychological, familial, sociological, or geographical condition potentially hampering compliance with the study protocol and follow-up schedule; those conditions should be discussed with the patient before registration in the trial.<br>- For women of childbearing potential, a serum pregnancy test will be done (and it must be negative), and an agreement to use a highly effective form of contraception during the study and at least the following 7 months will be obtained.<br>- Signed written informed consent obtained prior to any study-specific procedure.<br>- Completion of all necessary baseline surgical, laboratory, and imaging investigations prior to patient inclusion.	1. Patients with bone-only metastases.<br>2. Diffuse liver (≥50%) involvement on imaging.<br>3. Patients with brain metastasis as the sole site of metastatic disease and/or are symptomatic or require therapy to control symptoms. NB: Brain metastases are allowed, provided they are asymptomatic and/or controlled by previous radiotherapy. In case of recent prior brain radiotherapy, there must be evidence on MRI imaging of brain metastatic control for at least 6 weeks since the end of radiotherapy. Moreover, the patient should be at the end of corticosteroid therapy and be clinically asymptomatic.<br>4. Current uncontrolled hypertension despite medication intake (systolic 150 mmHg and/or diastolic 100 mmHg).<br>5. Current unstable angina.<br>6. History of symptomatic CHF of any New York Heart Association (NYHA) criteria or ventricular arrhythmia that requires treatment.<br>7. History of myocardial infarction within the last 6 months.<br>8. History of a decrease in LVEF to < 40% or symptomatic CHF with previous trastuzumab treatment.<br>9. Current dyspnea at rest due to complications of advanced malignancy, or other diseases that require continuous oxygen therapy.<br>10. Current severe, uncontrolled systemic disease (e.g., clinically significant cardiovascular, pulmonary, or metabolic disease; wound healing disorders; ulcers; or bone fractures).<br>11. History of other malignancy within the last 5 years, except for appropriately treated carcinoma in situ of the cervix, non-melanoma skin carcinoma, Stage I uterine cancer, or other cancers with a similar outcome as those previously mentioned.<br>12. Pregnant or lactating women.<br>13. Concurrent, serious, uncontrolled infections or current known infection with HIV, active hepatitis B, and/or hepatitis C.<br>14. Known prior severe hypersensitivity to trastuzumab.<br>15. Patient who received lapatinib within the 15 days prior to 89Zr-Trastuzumab injection.<br>16. Patient under a prohibited concomitant therapy, including vitamin K antagonist.<br>17. Patients with peripheral neuropathy Grade 3 or higher.

Supplementary Table 2.A. Relation between HER2 classification of tumor lesions and late metabolic response assessment

	Classification	n lesions	Metabolic lesion response after three T-DM1 cycles		PPV	NPV
			mR	mNR		
HER2 PET/CT	+	235	202	33	86%[1]	
	-	142	53	89		63%[2]

Exact 95% confidence intervals as follows: [1]: 81%-90%, [2]: 54%-71%.

Supplementary Table 2.B. Relation between early metabolic response of tumor lesions and anatomic response measurements and late metabolic response assessment

	Classification	n lesions	Anatomic lesion response after three T-DM1 cycles		PPV	NPV
			R	NR		
Early FDG PET/CT	mR	158	120	38	76%[1]	
	mNR	109	21	88		81%[2]
	Classification	n lesions	Metabolic lesion response after three T-DM1 cycles		PPV	NPV
			mR	mNR		
Early FDG PET/CT	mR	225	210	15	93%[3]	
	mNR	157	49	108		69%[4]

Exact 95% confidence intervals as follows: [1]: 68%-82%, [2]: 72%-88%, [3]: 89%-96%, [4]: 61%-76%.

Supplementary Table 2.C. Relation between the combination of HER2 classification and early metabolic response of tumor lesions, and anatomic response measurements and late metabolic response assessment

	Classification	n lesions	Anatomic lesion response after three T-DM1 cycles		PPV	NPV
			R	NR		
HER2 PET/CT / Early FDG PET/CT	+ / mR	130	108	22	83%[1]	
	+/ mNR	42	15	27		
	- / mR	28	12	16		
	- / mNR	65	6	59		91%[2]
	Classification	n lesions	Metabolic lesion response after three T-DM1 cycles		PPV	NPV
			mR	mNR		
HER2 PET/CT / Early FDG PET/CT	+ / mR	176	170	6	97%[3]	
	+ / mNR	59	32	27		
	- / mR	47	38	9		
	- / mNR	95	15	80		84%[4]

Exact 95% confidence intervals as follows: [1]: 74%-88%, [2]: 81%-97%, [3]: 93%-99%, [4]: 75%-91%.

n: number. mR: metabolically responding lesions. mNR: metabolically non-responding lesions. R: anatomically responding lesions. NR: anatomically non-responding lesions.

**Supplementary Table 3.** Accuracy data used in cost-effectiveness analysis	
Negative predictive value for HER2 PET/CT combined with early FDG PET/CT[1]	100%
Proportion of HER2-negative and early FDG non-responding patients[1]	24%
Proportion of HER2-negative patients[1]	32%
Cost of one cycle of T-DM1[2]	€3,700
Cost of one cycle of capecitabine + trastuzumab[2]	€1,070
Cost of one day outpatient care in a Belgian academic hospital	€1,500
Cost of HER2 PET/CT[1]	€3,000
Cost of FDG PET/CT[3]	€624.3

[1]Based on data from ZEPHIR study

[2]For a mean body weight of 70kg/mean body surface area of 1.6m^2, and based on the information from the Belgian Centre for Pharmacotherapeutic Information. https://www.bcfi.be/nl/start

[3]Based on reimbursement from Belgian National Institute for Health and Disability Insurance. https://www.riziv.fgov.be/nl/toepassingen/paginas/nomensoft.aspx

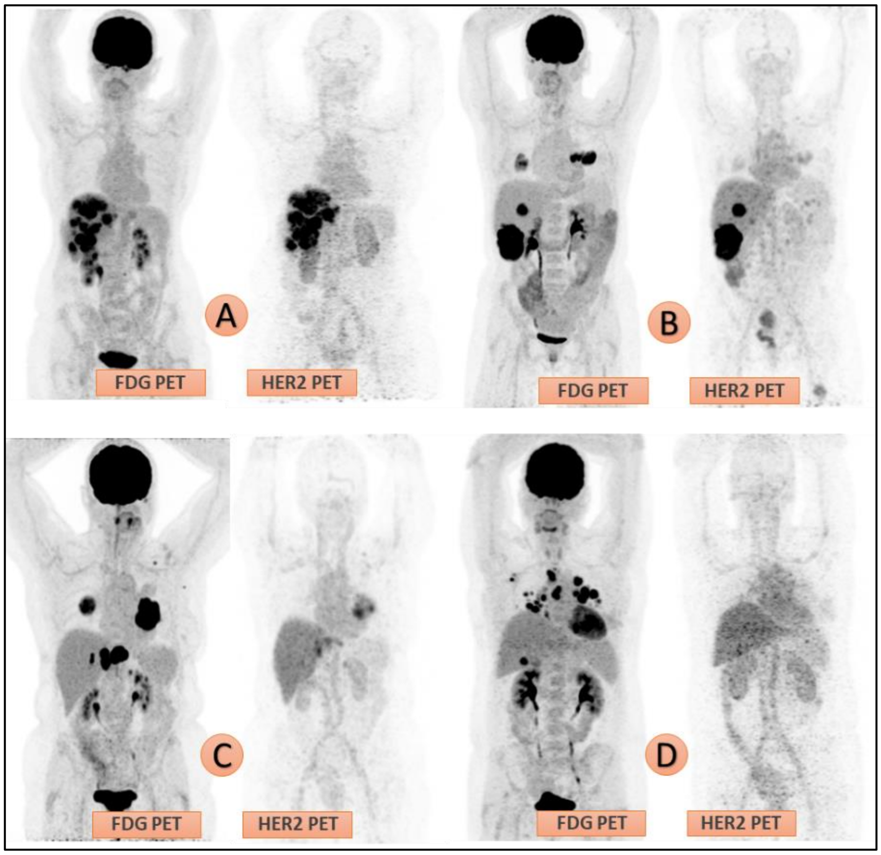

Supplementary Figure 1. Patterns of HER2 PET/CT confronted with FDG PET/CT, Maximum intensity projection images. Lesion uptake was considered pertinent when visually higher than surrounding background uptake. (A) Entire tumor load showed pertinent tracer uptake; (B) dominant part of tumor load showed tracer uptake; (C) minor part of tumor load showed tracer uptake; (D) entire tumor load lacked tracer uptake.

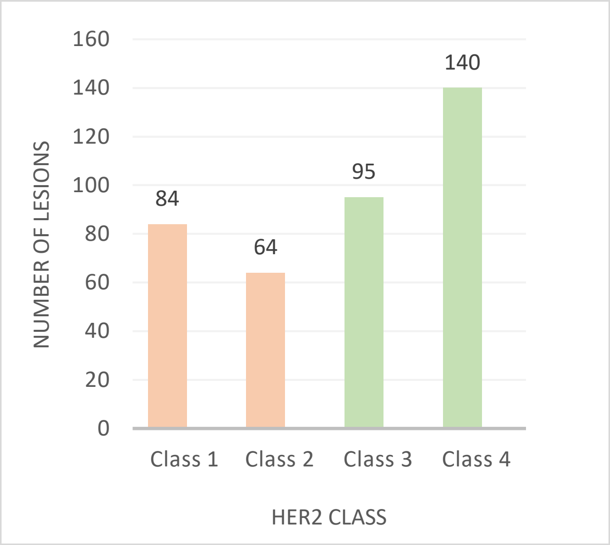

Supplementary Figure 2. Distribution of the target lesions among four HER2 classes based on a visual assessment of ^{89}Zr-trastuzumab uptake on the HER2 PET/CT. Class 1 and 2: HER2-negative lesions. Class 3 and 4: HER2 positive lesions.
